# First Ever Use of Proton Stereotactic Body Radiation Therapy Delivered with Curative Intent to Bilateral Synchronous Primary Renal Cell Carcinomas

**DOI:** 10.7759/cureus.1799

**Published:** 2017-10-24

**Authors:** Melissa A Frick, Arpit M Chhabra, Liyong Lin, Charles B Simone

**Affiliations:** 1 Department of Radiation Oncology, University of Pennsylvania; 2 Department of Radiation Oncology, University of Maryland Medical Center

**Keywords:** proton therapy, stereotactic, radiation therapy, ablative, inoperable, renal cell carcinoma

## Abstract

Limited therapeutic options exist for inoperable bilateral kidney tumors. We report the first ever use of proton therapy to treat primary renal cell carcinoma (RCC) and the first report of proton stereotactic body radiation therapy (SBRT) for RCC in an inoperable patient with synchronous RCCs treated with proton SBRT. The patient is a 47-year-old 450-pound female with multiple medical comorbidities, including Stage 3 chronic kidney disease (CKD), who was found to have bilateral renal masses during work-up for cellulitis and sepsis. Following resolution of her sepsis, subsequent cross-sectional imaging demonstrated interval growth of the left renal mass to 4.4 x 4.8 cm and the right renal mass to 2.0 x 2.6 cm. Bilateral biopsies were performed, with pathology revealing Furhman Grade 1-2 clear cell RCC on both sides. A customized SBRT plan delivered a total dose of 3,000 cGy in five fractions to the bilateral kidneys every other day using proton beam therapy. The patient experienced no grade > 1 acute adverse toxicities from proton therapy, and now at one year after treatment, she has had no clinical symptoms of late radiation-induced toxicities. Although a marginal decline in post-treatment glomerular filtration rate (GFR) was observed (baseline 34 mL/min/1.73m^2^, one-year post-SBRT 29 mL/min/1.73m^2^), the patient remains asymptomatic and without a requirement for intervention. In presenting a case in which proton SBRT was performed safely and effectively for a medically complex patient with inoperable synchronous bilateral RCC, we suggest that proton therapy is a promising therapeutic approach for even unilateral primary RCC where preservation of renal function is of importance and should be considered for medically inoperable patients. Further experience is needed to determine the optimal dose and fractionation regimen for renal SBRT with proton therapy.

## Introduction

Bilateral renal tumors are rare, accounting for only 4% of patients with renal cell carcinoma (RCC). Surgical resection is the standard curative treatment for bilateral RCC and delivers outcomes comparable to that of unilateral RCC [1]. Many patients, however, are not surgical candidates due to technical limitations, medical comorbidities, and/or an inability to preserve normal kidney function - a particular challenge for bilateral cases. While radiofrequency ablation and cryoablation can be considered in select inoperable cases, their deliverability and efficacy are limited by tumor size and location.

Conventional radiotherapy has been limited as a modality, given the relative radio-resistance of RCC and the risks of radiation-induced toxicities present with irradiating large volumes of the kidney. Stereotactic body radiation therapy (SBRT), also known as stereotactic ablative radiotherapy (SABR), however, may overcome these limitations by taking advantage of highly conformal dose delivery, image guidance, and rapid dose fall-off. This allows delivery of ablative doses to the tumor while sparing normal tissues at risk [2]. Large fractional doses have been shown to increase the biological effectiveness of radiation against RCC, likely by acting on a different set of biological mechanisms than those leveraged by conventional RT [3]. We were recently the first group to demonstrate that photon-based SBRT can be safe and effective for the treatment of renal metastases and allow for prolonged local control and overall survival in patients with oligometastatic disease [4].

In patients with pre-existing kidney disease, however, incidental irradiation of the uninvolved kidney with photon-based SBRT presents a high risk of kidney failure requiring chronic hemodialysis with resulting impairment of quality of life and decreased overall survival. This risk is magnified in patients with bilateral renal malignancies. Herein exists an opportunity where proton beam radiotherapy could offer a dosimetric advantage due to its characteristic Bragg peak and lack of exit dose. Minimization of dose to the uninvolved kidney and surrounding tissues could substantially improve clinical outcomes and reduce life-threatening toxicities when compared to plans delivered with photon therapy. 

For the first time, we herein report the safety and efficacy of using proton SBRT in a patient with inoperable bilateral synchronous RCC.

## Case presentation

History and physical

A 47-year-old 450-pound female with multiple comorbidities presented to our department for outpatient consultation with bilateral synchronous Stage I RCCs for consideration of external beam radiation therapy. Her bilateral renal masses were incidentally found during work-up for methicillin-resistant Staphylococcus aureus cellulitis and sepsis. Following recovery of her sepsis, she underwent repeat abdominal magnetic resonance imaging (MRI) that demonstrated interval growth of the left-sided mass to 4.4 x 4.8 cm and of the right-sided mass to 2.0 x 2.6 cm. The patient underwent image-guided biopsies of the bilateral masses, both of which revealed Furhman Grade 1-2 clear cell RCC. She was determined to be a poor surgical candidate due to her extensive comorbidities, including morbid obesity with a body mass index (BMI) > 90, pulmonary hypertension, chronic obstructive pulmonary disease, congestive heart failure, peripheral vascular disease, Stage 3 chronic kidney disease (CKD), adrenal insufficiency, obstructive sleep apnea, and recurrent wound infections. She was referred by her urologist to an outside radiation oncology practice but was not offered therapy due to concerns of excessive dose and resultant risk of toxicity to her remaining normal kidney tissue. She was referred to our institution for consideration of proton therapy, given its potential dosimetric advantages and the ability of our proton treatment couch to accommodate the patient’s weight.

At the time of consultation, the patient was asymptomatic from her RCCs; however, her performance status was limited due to her underlying comorbidities. The patient required 3 liters of oxygen via nasal cannula, even while at rest in her wheelchair. Her mobility was limited to transfer from wheelchair to bed, and she was unable to walk more than a few steps, even with a walker. Her immobilization resulted in the development of recurrent pressure ulcers, the most significant of which required multiple incisions, drainages, debridements, and admissions for bacteremia and sepsis.

Her previous surgical history included a Caesarean section, laparoscopic cholecystectomy, and multiple exploratory laparotomies. Her family history was notable for uterine and breast cancer in her mother and maternal aunt, respectively. She was a previous smoker of > 50 pack years, and she had no history of alcohol or intravenous (IV) drug use. Her exam revealed morbid obesity, distant heart sounds, 1+ bilateral pitting edema, damp desquamation in her skin folds at the flanks without ulceration, oxygen use, and an Eastern Cooperative Oncology Group (ECOG)  performance status of 3+. Her creatinine levels were stable at 1.62 mg/dL and her estimated glomerular filtration rate (GFR) was 34 mL/mg/1.73m^2^.

Patient setup and treatment plan

**Figure 1:**
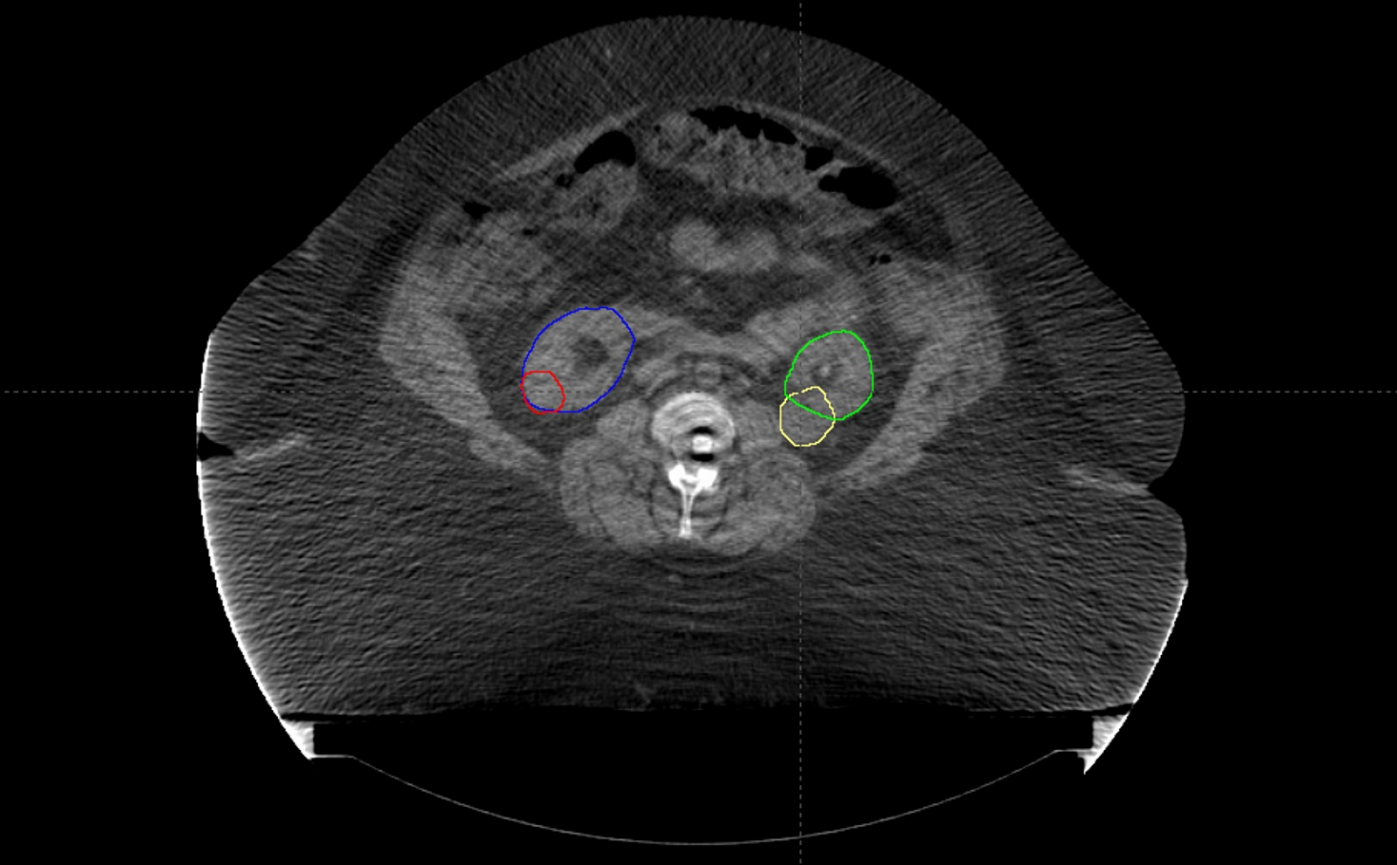
Pre-treatment Disease Extent Axial slice of the CT image from the radiation planning simulation depicting bilateral synchronous renal cell carcinomas. The gross target volume for the right and left renal masses are contoured in red and yellow, respectively. The right and left normal kidney volumes are contoured in blue and green, respectively.

The patient underwent simulation in the supine position with her arms above her head; a full body vac-lock bag was used for immobilization. To assess and account for tumor motion, a four-dimensional (4D) computed tomography (CT) scan was performed at the time of simulation to monitor the location of the tumor during all phases of the respiratory cycle (Figure 1).

**Figure 2:**
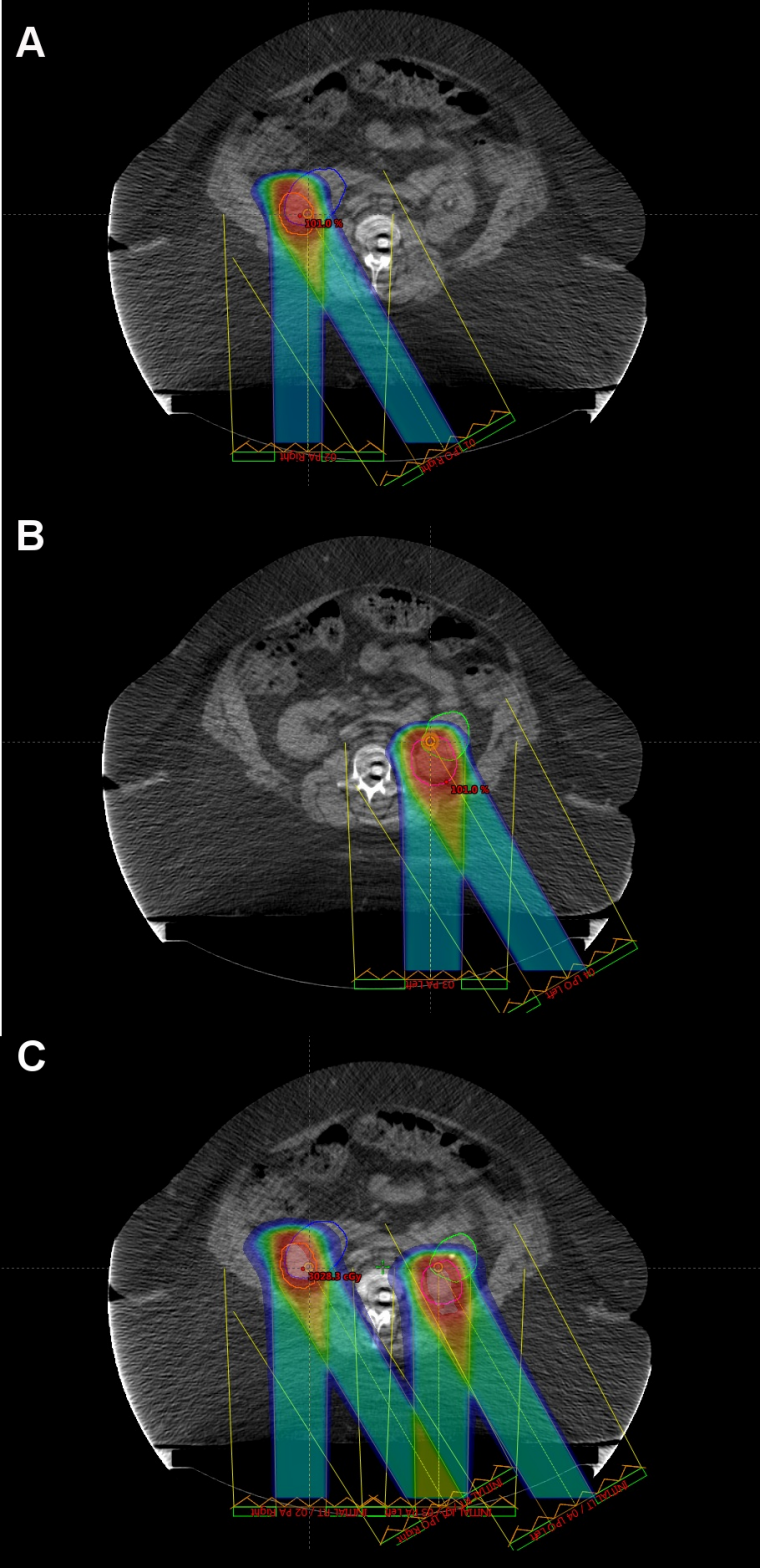
Proton Stereotactic Body Radiation Therapy Plans The proton treatment plan is displayed for the A) right renal target volume and B) left renal target volume, with lesions treated with two separate isocenters. Treatment fields are displayed, with each volume being treated with posterior and left posterior oblique beams. The right planning target volume is contoured in orange, whereas the left planning target volume is contoured in magenta. The radiation dose is displayed as a color wash. Color coding: red = 100% to blue = 10% of 30 Gy in 6 Gy fractions. C) A plan summary of the right and left treatment courses is depicted.

**Figure 3:**
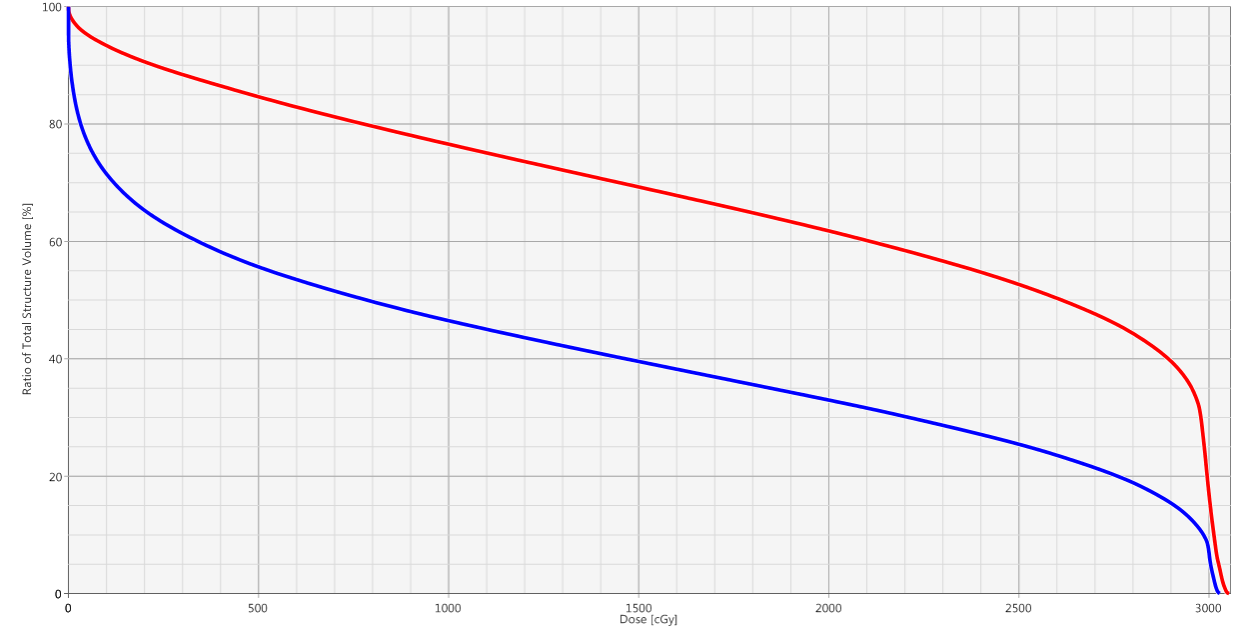
Dose-Volume Histogram Dose-volume histogram demonstrating radiation dose delivered to right (blue) and left (red) kidneys.

Given that the patient was not a candidate for IV contrast at the time of simulation due to her preexisting CKD, she underwent a 4D positron emission tomography (PET)/CT simulation to aid in target volume delineation. The bilateral renal masses and organs at risk were contoured using the simulation CT. Gross tumor volume (GTV) was defined as the radiographically visible tumor based on the CT/PET simulation images and fused MRI abdominal imaging. Bilateral internal gross tumor volumes (iGTVs) were created that represented an expansion of the GTV to account for tumor motion, as assessed by the 4D simulation imaging. Beam-specific planning treatment volumes (PTVs) were created for each tumor that were approximately a 5 mm expansion on the iGTVs. Customized SBRT plans using separate isocenters were designed to deliver a total dose of 3,000 cGy in five fractions to the bilateral kidney target volumes delivered every other day (Figure 2). Plans were prescribed to the 100% isodose line so as to avoid hot spots sometimes employed with SBRT in order to minimize high doses delivered to normal kidney tissues immediately adjacent to the target volume. Two fields were used for each isocenter. Posterior-oriented beam arrangements were determined to be most robust. Right posterior oblique angles were not possible for the right isocenter since her body habitus was such that her entire abdominal girth was not able to fit within the CT field of view. As a result, posteroanterior (PA) and left posterior oblique (LPO) beam arrangements were used for both targets, and fields were intentionally widely separated to minimize skin dose in light of her history of chronic pressure ulcers. As such, conformity was sacrificed and two-field plans were selected as a compromise in order to minimize normal kidney and skin doses. Plans were designed to deliver 30 Gy (100% of the prescription dose) to at least 95% of each PTV and 27 Gy (90% of the prescription dose) to at least 99% of each PTV. The normal tissue constraints included the hottest 200 cc (D200cc) of total kidney volume receiving < 17.5 Gy, small bowel maximum point dose (DMax) < 20 Gy, spinal cord DMax < 22 Gy, skin DMax < 20 Gy, and liver mean dose < 12 Gy. No dose constraint was applied to the adrenal glands in an attempt to prioritize renal sparing. All tumor volume and normal tissue constraints were met. The plan included a minimal overlap of fields with a less than 2.0 cm x 2.0 cm area of posterior skin receiving a half-max dose of > 16.5 Gy (peak dose = 22 Gy) (Figure 2C). Of note, D200cc of her total kidney was 16.5 Gy, her right kidney mean dose was 12.2 Gy, and her left kidney mean dose was 20.6 Gy (Figure 3).

The patient underwent daily pre-treatment kV-KV and cone-beam CT (CBCT) scans in the treatment position prior to each fraction to ensure optimal target localization, although CBCT image quality was suboptimal given the patient’s body habitus.

Acute toxicities

Acute toxicities were minimal and included Grade 1 urinary urgency, Grade 1 urinary incontinence, and Grade 1 fatigue; she had no hematuria, flank pain, nausea, changes in bowel habits, loss of appetite, or other toxicities during or in the weeks and first months following proton SBRT. Her performance status remained stable at ECOG 3 by the completion of treatment and at one month following treatment.

Post-treatment follow-up

**Figure 4:**
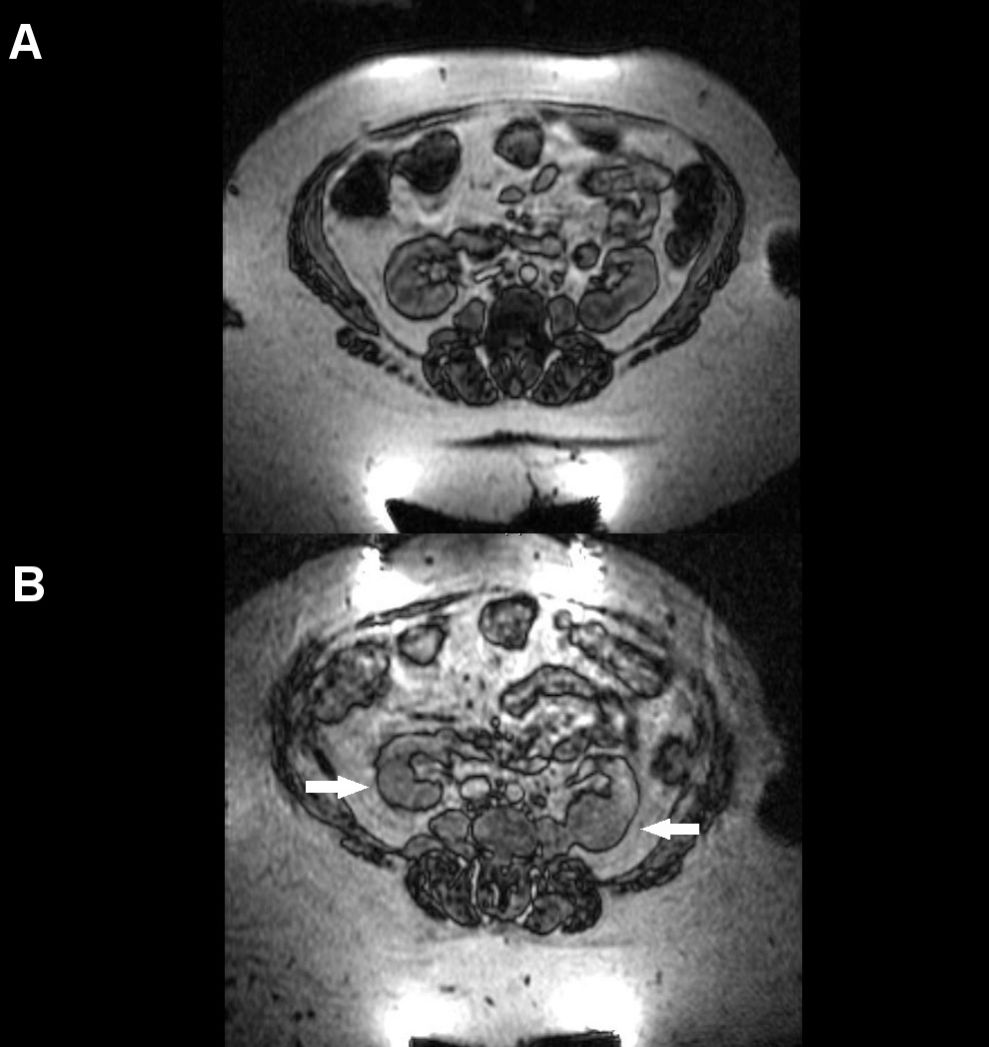
Post-treatment Imaging Post-treatment abdominal MRIs are depicted at A) three months and B) 12 months following the completion of proton stereotactic body radiation therapy. Bilateral lesion stability, indicated by small arrows, is evident.

Three months following radiation treatment, the patient reported resolution of both her fatigue and urinary symptoms to pre-treatment levels, and she had no additional complaints. An MRI at this time demonstrated stable appearing bilateral renal masses, now 4.0 x 4.3 cm on the right and 3.0 x 2.7 cm on the left, consistent with early findings of a delayed response to treatment (Figure 4A). She denied new symptoms at her six month and nine-month follow-up visits. At her one-year follow-up visit (her most recently completed visit), the patient continued to do well with no evidence of renal failure or any other reported late toxicities associated with proton SBRT. An MRI at that time demonstrated stable findings, with the left-sided lesion measuring 4.6 x 4.4 cm and the right-sided lesion measuring 2.3 x 2.6 cm (Figure 4B). While her creatinine level has marginally increased over the past year from the pre-treatment baseline of 1.62 mg/dL to 2.00 mg/dL (estimated GFR = 29 mL/min/1.73m^2^, previously 34 mL/min/1.73m^2^), the patient remains asymptomatic with no significant clinical toxicity.

## Discussion

To the best of our knowledge, this is the first reported case of primary RCC treated with proton therapy. This also is the first report of delivering SBRT with protons for RCC and the first report of proton therapy to treat bilateral synchronous renal malignancies. The novelty of this treatment regimen was commanded by the particular set of clinical dilemmas with which our patient presented: a body habitus posing technical limitations for photon-based delivery, multiple comorbidities precluding standard-of-care surgical intervention, and bilateral tumors in the setting of underlying CKD, placing paramount importance on the preservation of her renal function. With this case, we demonstrate that proton SBRT is feasible, safe, and well-tolerated, providing a rationale to consider extending the use of this modality for medically inoperable unilateral RCC, particularly for complex patients and/or where renal function preservation is critical.

A limited number of retrospective and phase I/II studies have demonstrated excellent local control for primary RCC using photon-based SBRT. In 2012, Siva, et al. conducted a systematic review of 10 studies (three prospective and seven retrospective) with 126 patients treated with SBRT for inoperable RCC [5]. These studies utilized a wide range of doses, fractionation schedules, and techniques, with the most commonly described doses being 30 to 45 Gy in 3 to 5 fractions. Local control rates ranged from 84% to 100% with a weighted Grade 3+ toxicity rate of 3.8%.

Since that review, several additional studies have been published and continue to support the safety and efficacy of kidney SBRT with photon therapy. The University Hospitals Seidman Cancer Center in Cleveland reported on their Phase I dose-escalation study with 19 patients receiving 24 to 48 Gy in four fractions. They described a limited rate of early radiation-related side effects: one Grade 4 acute cardiac event (not thought to be treatment-related), one Grade 4 acute duodenal ulcer (possibly treatment-related), and no Grade 3 adverse events. Of 15 patients with evaluable responses, three had a partial response and 12 had stable disease [6]. A group from the Munich CyberKnife Center reported on their prospective study including 40 patients with RCC and upper tract transitional cell carcinomas treated with single fraction (25 Gy) robotic stereotactic radiosurgery. No significant toxicities were seen at a median follow-up of 28.1 months. Of the 45 renal tumors treated, they observed a measurable size reduction in 38 lesions with 19 undergoing complete responses [7]. Most recently, investigators from the Peter MacCallum Cancer Centre in Melbourne published updated results from their prospective study delivering 26 Gy in one fraction or 42 Gy in three fractions. Thirty-three patients with 34 tumors were treated with linac-based SBRT, and only one Grade 3 toxicity (fatigue) was reported, with no Grade 4-5 toxicities observed. At a median follow-up of 24 months, four patients had partial responses and 28 had stable disease (unpublished data, in press at BJU Int, 2017). In light of these promising early results of photon-based SBRT in the treatment of primary inoperable RCC, the International Radiosurgery Oncology Consortium for Kidney (IROCK) recently released its consensus statement describing treatment indications, dose constraints, delivery techniques, and response assessments [8].

Balancing the need for maximal oncologic control and the chance for a cure against the goal of maximal functional preservation is of particular importance for bilateral renal tumors, and this is a current clinical challenge for photon-based radiotherapy. The need to reduce incidental irradiation to the uninvolved kidney tissue is of critical importance in patients with bilateral RCCs, especially in the setting of pre-existing CKD as was the case for our patient. Such challenging cases are where the dosimetric advantages of proton radiotherapy compared to photon-based modalities are magnified. Photons deposit their peak dose shortly after entering tissue and experience an exponential decrease of deposited dose with increasing depth. In comparison, protons travel through tissue with limited dose deposition until reaching the end of their path, where they deposit a majority of their energy, termed the “Bragg peak,” and thereafter have essentially zero energy beyond that depth, resulting in greater sparing of normal tissues [9]. Although there is a single report in the literature on the use of non-SBRT delivery of carbon ions – another form of particle therapy – for the treatment of kidney malignancies, the use of proton radiotherapy for treatment of kidney cancer has not previously been described. Nomiya, et al. treated 10 primary RCC patients with carbon ion radiotherapy to a median dose of 72 Gy in 16 fractions. At a median follow-up of 57.5 months, the five-year local control, progression-free survival, and overall survival were 100%, 100%, and 74%, respectively. One Grade 4 late toxicity of the skin was observed and renal function was generally well preserved, except in the patients with diabetic nephropathy. Notably, in keeping with the radiographic findings following treatment in our present patient, these investigators observed that most tumors showed no change or a transient enlargement in volume for several months after particle therapy treatment but then displayed a very slow shrinkage pattern over the long-term with one tumor still shrinking after nine years [10].

Taken together, given the dosimetric benefits of proton therapy and the potentially curable nature of the patient’s synchronous tumors, we offered definitive proton SBRT to her bilateral tumors to maximize the biologically effective dose and the likelihood of long-term control while preserving her normal kidney parenchyma and minimizing skin dose. This treatment, however, was not without risks, particularly given her pre-existing CKD. The patient experienced no severe acute adverse events and now, at one year following proton SBRT, she has at least stable disease without the development of any late symptoms from treatment. Although she experienced a marginal decline in post-treatment GFR, the patient remains asymptomatic and without a requirement for intervention.

Of note, we elected not to apply a dose constraint on the adrenal glands in our treatment plan since there is no well-established dose constraint for these organs at present and our priority in treatment was to minimize dose to her bilateral kidneys. The patient was referred to an endocrinology specialist prior to treatment, and she will continue to undergo surveillance lab work and potential replacement therapy should she develop a late endocrine deficiency from treatment. Fortunately, however, given the specific location of her lesions, her adrenal glands were significantly spared of collateral irradiation with proton beam radiotherapy.

In this case report, we are limited by relatively short follow-up, especially as the patient will remain at risk for late renal toxicity for years following treatment. Additionally, despite early encouraging radiographic stability over serial post-treatment imaging, which is the most likely radiographic finding in patients with durable treatment responses following SBRT, we cannot definitely confirm a lack of viable residual tumor since a histologic confirmation of treatment response was not sought. Although radiological response assessment after ablative doses of radiation therapy is, at times, difficult to interpret, given the lack of radiographic enlargement and her medical comorbidities, biopsy at this time is not felt to be medically indicated. We were also limited in field design by her specific tumor locations and bilateral lesions, as well as her body habitus. It is likely that conformity can be optimized further for future patients who are treated with this potentially beneficial modality through different field designs and using more than two beams for each lesion. Despite these various limitations, this case report presents the feasibility of proton SBRT in inoperable primary RCC and should be used as support by other practitioners.

## Conclusions

We present the first application of proton SBRT in the treatment of synchronous bilateral RCC and provide early evidence that proton SBRT is feasible, efficacious, and associated with minimal toxicities. Proton SBRT should be considered as an emerging treatment modality for patients with inoperable primary RCC where preservation of renal function is of paramount importance, including those with pre-existing CKD, bilateral lesions, only one kidney, or particularly large tumors. Further experience is needed to determine a dose and fractionation regimen that optimizes both local control and safety.
